# Parenting Styles, Feeding Styles, Feeding Practices, and Weight Status in 4–12 Year-Old Children: A Systematic Review of the Literature

**DOI:** 10.3389/fpsyg.2015.01849

**Published:** 2015-12-14

**Authors:** Netalie Shloim, Lisa R. Edelson, Nathalie Martin, Marion M. Hetherington

**Affiliations:** ^1^Biopsychology Group: Human Nutrition, School of Psychology, Institute of Psychological Sciences, University of LeedsLeeds, UK; ^2^Behavior and Perception, Nestlé Research CenterLausanne, Switzerland

**Keywords:** parenting styles, feeding styles, feeding practices, obesity, children

## Abstract

Childhood is a critical period in the development of obesity. Eating patterns established early in life track into later life. Therefore, parental approaches to feeding in their general parenting style, feeding styles, and specific feeding practices will have a profound impact on how children eat and grow. A systematic research review following PRISMA guidelines was conducted to identify, discuss and integrate recent research investigating the relationship between parenting styles, feeding styles, feeding practices, and body mass index (BMI) in children. Medline (Ovid), PsycINFO, Web of Science, and Food Science and Technology Abstracts were systematically searched using sensitive search strategies. Studies were limited to papers published in English between 2010 and February 2015 with participants aged 4–12 years old with outcomes including obesity, change in weight, or BMI. The search yielded 31 relevant quantitative peer-reviewed papers meeting all inclusion criteria: seven longitudinal, 23 cross-sectional, one randomized control trial. Associations between parenting style and child BMI were strongest and most consistent within the longitudinal studies. Uninvolved, indulgent or highly protective parenting was associated with higher child BMI, whereas authoritative parenting was associated with a healthy BMI. Similarly for feeding styles, indulgent feeding was consistently associated with risk of obesity within cross-sectional studies. Specific feeding practices such as restriction and pressure to eat were linked to BMI, especially within cross-sectional studies. Where child traits were measured, the feeding practice appeared to be responsive to the child, therefore restriction was applied to children with a high BMI and pressure to eat applied to children with a lower BMI. Behaviors and styles that are specific to the feeding context are consistently associated with child BMI. However, since obesity emerges over time, it is through longitudinal, carefully measured (through questionnaire and observation) studies which take account of child appetite and temperament that the association between parenting style, feeding style, specific feeding practices, and child obesity will be understood.

## Introduction

Childhood obesity has become a major global health challenge. In developed nations, approximately 24% of children and adolescents are overweight or obese and in developing countries, rates have reached 13% (from 8% in 1980; Ng et al., 2014). While obesity rates may have leveled off or declined in some nations (Ogden et al., 2014), prevalence remains high. Obese children are likely to become obese adults and are at higher risk of developing a range of chronic diseases such as cardiovascular disease (CVD), type 2 diabetes and cancer (Lloyd et al., 2012). Therefore, it is important to understand the factors which influence obesity development in order to guide future research, interventions, and policy.

Obesity is caused by the chronic mismatch between energy intake and expenditure, with food intake in excess of requirements increasing body mass index (BMI). This chronic imbalance is influenced by gene-environment interactions (Hetherington and Cecil, 2010). Some of these factors, including candidate genes, socio-economic status (SES), exercise, sedentary behavior, and sleep are well established (Hetherington and Cecil, 2010; Craigie et al., 2011; Hart et al., 2011; Wang et al., 2011; Ho et al., 2012; Magee and Hale, 2012). The interaction between parents and their children is crucial since this is when intake and activity patterns are established. In turn, these influence children's growth patterns and risk of becoming overweight. It is important to understand the interplay between parenting, the food environment, and child weight outcomes. The current review examines the specific modifiable aspects of parent-child interactions (parenting/feeding styles and feeding practices) and their associations with child BMI. Modifiable aspects are of interest because this permits consideration of future interventions to prevent or treat obesity.

Parents are important agents through which food preferences and intake patterns are set, via both direct and indirect influences, from controlling the child's intake to passively modeling a healthy or unhealthy diet (Birch and Fisher, 1998; Brown and Ogden, 2004; Cooke et al., 2004; Clark et al., 2007; Beydoun and Wang, 2009; Larsen et al., 2015). In the present review, the focus is parenting or feeding styles and specific feeding practices which might influence child BMI.

Parenting style is a general behavioral construct which sets the emotional context within which parents and children interact. These have often been characterized as having at least two dimensions: demandingness (how much control parents exercise) and responsiveness (warmth and acceptance in response to their children's needs). Within this definition, there are four types of parenting styles, varying along these two dimensions: (1) ***authoritative parenting***, associated with a high level of demandingness and rules with high responsiveness to the child; (2) ***authoritarian parenting*** linked to high demandingness but low responsiveness characterized by rules but with less influence from the child's needs; (3) ***indulgent parenting*** combining low demandingness and high responsiveness with few rules but high engagement with the child's needs; and (4) ***uninvolved parenting*** which is associated with both low demandingness and low responsiveness. An authoritative parenting style is generally associated with the most positive child outcomes, such as higher school performance (Maccoby and Martin, 1983; Darling and Steinberg, 1993) and with a more positive home food environment (Johnson et al., 2012).

**Figure 1:**
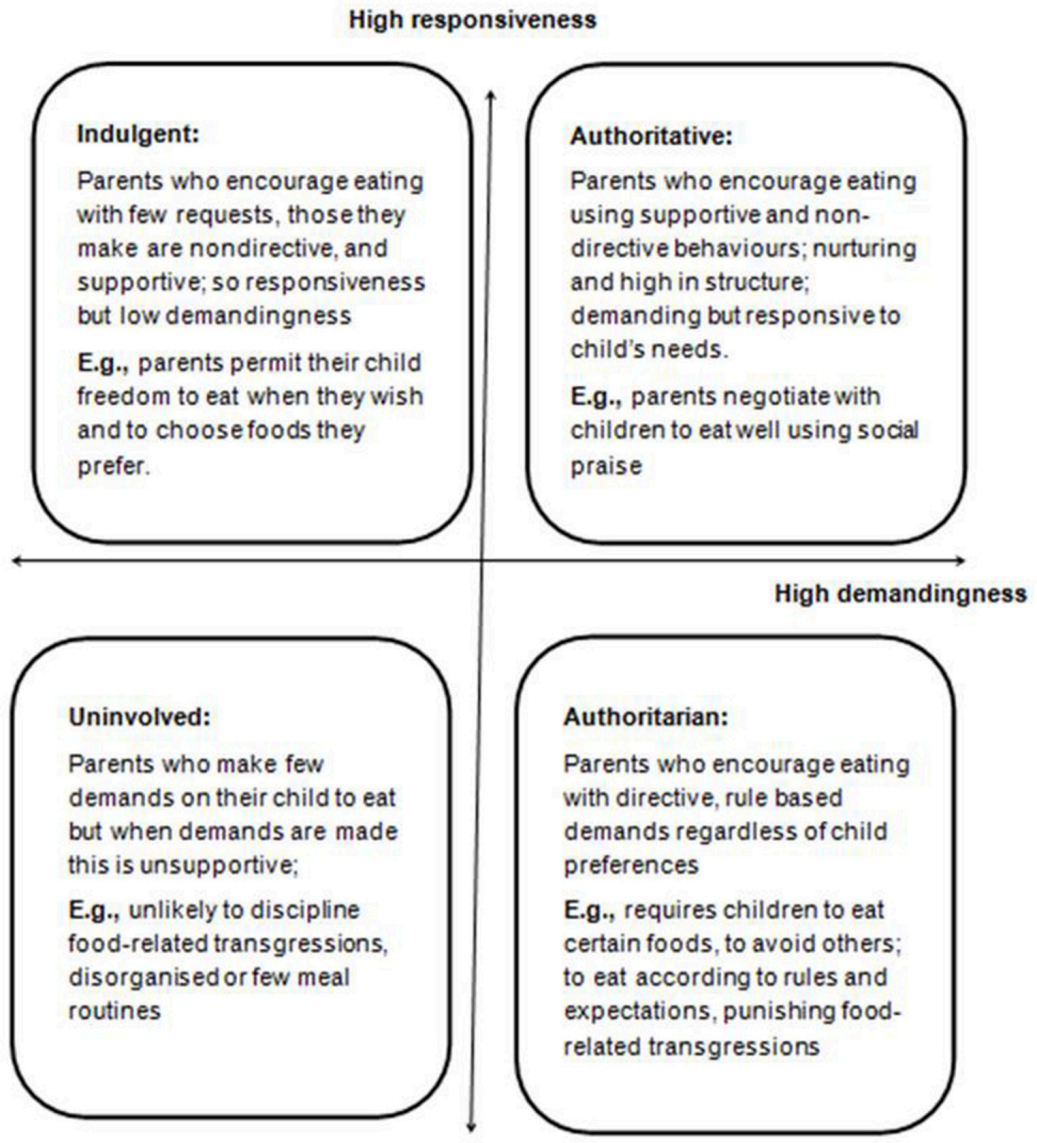
**Summary of the parental feeding styles adapted from typological approach to parenting developed by Hughes et al. (2005)**.

Feeding styles may be viewed as a sub-category of parenting styles that are specific to mealtimes and therefore the same dimensions of demandingness and responsiveness are applied in the feeding context (Hughes et al., 2005; Ventura and Birch, 2008; Blissett, 2011). Thus, with an ***authoritative*** feeding style, parents actively encourage their child to eat but achieve this through supportive behaviors including rules explained in a sensitive way whereas with an ***authoritarian*** feeding style, parents encourage eating through parent-centric rules (Figure 1).

*Feeding practices* refer to the specific goal-directed behaviors used by parents to directly influence their children's eating. This might include attempts to increase or decrease intake of certain foods. Common feeding practices include: modeling eating behaviors, restricting certain types of food, pressuring children to eat, rewarding positive behaviors with food, and availability of food at home. A more comprehensive list of specific feeding practices can be found in Gevers et al. (2014).

Given the complexity of the family meal structure, parenting style, feeding style, and feeding practices are defined within broad constructs to simplify research and to promote understanding. The general ways in which parents interact with their children (parenting style) and particularly during meals and snack times (feeding style) may influence parents' choice of feeding practices or the outcomes of these practices (Stang and Loth, 2011; Collins et al., 2014; Larsen et al., 2015). The interaction between parents and children at the family meal is bi-directional and informed by a number of different environmental factors (e.g., income and culture), parental attributes (beliefs, attitudes, behaviors), and child characteristics (temperament, eating traits and learned behaviors). These additional factors will be taken into account in the current review, where available.

The present systematic review was undertaken to identify, discuss, and integrate recent research exploring the specific parenting styles, feeding styles, and feeding practices which increase or decrease the risk of childhood obesity in children aged 4–12 years old. The search was limited to studies published in the last 5 years to provide an update since the reviews on parenting/feeding styles published by Vollmer and Mobley (2013) and on feeding practices by Hurley et al. (2011) cover only articles published up until 2012 and 2009, respectively. Another goal of the current review was to identify the gaps in the existing literature so that recommendations for future research and interventions can be made. The review structure was based on the Preferred Reporting Items for Systematic Reviews and Meta-Analyses guidance (PRISMA, Moher et al., 2009).

## Methods

A systematic search was conducted to identify relevant studies investigating associations between parenting styles, feeding styles, feeding practices, and obesity/overweight in 4–12 year-old children. The search was conducted using Medline (Ovid), PsycINFO, Web of Science, and Food Science and Technology Abstracts. The search strategy (the comprehensive search strategy is available from the authors upon request) addressed key search terms for obesity, parenting styles, and feeding behaviors (including styles and practices). More specifically, literature searches were conducted using a combination of the following key terms: parenting (style), feeding (style, practices, behaviors), and weight-related keywords: eating, diet, (over)weight, obesity, BMI. Age-related terms such as child were not included in the search phase to avoid missing studies that used unusual terms (e.g., third graders) or that did not include age terms in the title of the paper. Nevertheless, studies were limited by child age at a later stage. The final search was limited to papers published in English between 2010 and February 2015, a period of just over 5 years, permitting recent and timely evidence to be included within the search. Studies were included in the review if the mean age of the participants was between 4 and 12 years old and therefore studies with participants with an overlapping age range but with a mean age of < 4 or >12 were excluded. For longitudinal or intervention studies, the sample age at baseline was used. This age group provided a broad base to explore risk within a time of childhood when there is more independence than during infancy and pre-school but with a greater dependence on families for most meal occasions than adolescents or young adults. Studies of children with co-morbidity such as eating disorders, diabetes or other diseases were excluded. Studies in which the outcome was not child BMI or change in weight were excluded as well. Four review papers (Thompson, 2010; Hurley et al., 2011; Sleddens et al., 2011; Vollmer and Mobley, 2013) were identified and are addressed in the discussion of this paper. Titles and abstracts were screened for relevance and content. If the abstract did not provide sufficient data, the entire paper was read to determine whether it met the inclusion criteria. Publications which included multiple age groups or which addressed several independent measures were fully read but only findings that met the inclusion criteria are addressed in this review. The included papers (*N* = 31) were then summarized in a table covering the main characteristics of each paper (Table 1).

**Table 1:** **Characteristics of the papers identified in the review**.

**Parenting Styles**	**Country**	**Sample size; mean (SD) or age range of children in years**	**Study design**	**Validated questionnaires**	**Other measures**	**Main findings**	**Scores**
Hancock et al., 2014	Australia	2596 parents and children; 4–5	Longitudinal	Protective parenting measure	Child weight and height; demographics	Protectiveness at age 6–7 not associated with weight at any age. No association between maternal protectiveness at age 8–9 and overweight/obesity at age 4–5, 6–7 or 8–9 years. Greater maternal protectiveness at age 8–9 was associated with children being overweight or obese at ages 10–11	17
Olvera and Power, 2010	USA	69 mothers and children; 6.7(1.3)	Longitudinal: 3 year follow-up	PDI	Child and parent weight and height; demographics	Indulgent mothers were more likely to have overweight children in a 3 year follow up compared to authoritative or authoritarian mothers	18
Rodenburg et al., 2011	Netherlands	1665 parents and children; 8–9	Cross-sectional	Home Environment Survey (HES); FFQ; Dutch Youth Health Care questionnaire; Parenting scales (Steinberg et al., 1989; Dutch translation)	Child and parent weight and height; demographics	Rejecting parenting style was associated with increased child BMI	20
Rodenburg et al., 2012	Netherlands	1275 parents and children; 8.2(0.5)	Longitudinal	CEBQ; FFQ; Parenting scales (Steinberg et al., 1989; Dutch translation)	Child's weight and height.	Authoritative parenting was positively associated with fruit consumption of fussy eaters. Neglecting parenting showed a positive association with child's weight	20
**FEEDING STYLES**							
Frankel et al., 2014	USA	296 parents and children; 4.4(0.7)	Cross-sectional	CFSQ; CEBQ	Child and parent weight and height	Indulgent feeding style was negatively associated with satiety responsiveness and positively associated with enjoyment of food and BMI	18
Hennessy et al., 2010	USA	99 parents and children; 9(1.5)	Cross-sectional	PDI-S; CFSQ; CFQ	Child's weight and height; demographics.	Feeding style, rather than parenting style correlated with child BMI. Indulgent feeding style was associated with a higher child BMI z-score; feeding style moderated the link between restriction and child BMI z-score	18
Hughes et al., 2011	USA	177 parents and children; 4.4(0.7)	Cross-sectional with repeated behavioral observations	CFSQ	Child's weight and height; demographics; Home Observation Coding System; Feeding Behavior Coding System (FBCS)	Parents with indulgent feeding styles made fewer demands on their children to eat during dinner and showed lower levels of negative affect and intrusiveness. Hispanic boys with indulgent parents had significantly higher BMI z scores compared to Hispanic boys in the other three feeding style groups. No differences in any other ethinic group	19
Tovar et al., 2012	USA	383 mothers and children; 6.2(2.7)	Cross-sectional baseline data from a RCT	CFSQ; Centre for Epidemiologic Studies Depression Scale (CES-D); Perceived Stress Scale (PSS)	Parent and child weight and height; demographics	Feeding styles significantly differed by child BMI z-score. Indulgent feeding style was associated with a higher child weight	16
Yilmaz et al., 2013	Turkey	380 parents and children; 5–7	Cross-sectional	PFSQ	Child weight and height; demographics; visual scale of child body shape	Misperception of child weight was common (42%). Emotional feeding and encouragement to eat were lower in children perceived to be overweight. Emotional feeding and permissive control were lower in children correctly categorized as overweight	11
**FEEDING PRACTICES**							
Blissett and Bennett, 2013	UK and Germany	171 families; 2–12	Cross-sectional and cross-cultural comparison	CFPQ; CEBQ; Dutch Eating Behavior Questionnaire (DEBQ)	Parent's and child weight and height; demographics	Black Afro-Caribbean (BAC) families in the UK followed a more restrictive feeding regime. White German parents used the lowest levels of pressure to eat. Parental restriction was positively associated with child BMI in BAC families only	17
Campbell et al., 2010	Australia	392 parents and children; 5–6, 10–12	Longitudinal: 3 year follow-up	CFQ (Restriction)	Parent's and child's weight and height; demographics	Higher baseline restriction scores were associated with lower BMI z-scores at 3 year follow up in the younger (5–6 years) but not older (10–12 years) children	16
Cardel et al., 2012	USA	267 children; 7–12	Cross sectional	CFQ	Child's weight and height; Pubertal status; Genetic admixture; Socioeconomic status	Parental restriction was a significant predictor of child's BMI. Parental pressure to eat was negatively associated with child BMI	21
Costa et al., 2011	Brazil	110 parents and children; 6–10	Cross sectional	CFQ; The Brazil Economic Classification Criterion	Child weight and height	Boys were more likely to be overweight or obese than girls; high child BMI was associated with higher perceived weight, concern, monitoring and restriction. Pressure to eat was inversely associated with child BMI	14
Dev et al., 2013	USA	329 parents and children; 2–5	Cross-sectional data from a longitudinal study	CFQ;Three Factor Eating Questionnaire (TFEQ); Eating Attitudes Test (EAT)	Child weight and height; 22 previously identified risk factors for obesity.	Restriction linked to greater likelihood to have an overweight/obese child	13
Gubbels et al., 2011	Netherlands	2021 mothers and children; 5–7	Longitudinal	CFQ; FFQ	Parent and child's weight and height; child's energy balance-related parenting practices; child's dietary intake, activity behavior; demographics	Restriction of unhealthy food was positively associated with child's BMI; monitoring a child's diet was positively associated with children's diet quality	15
Holland et al., 2014	USA	170 parents and children; 7–11	Cross-sectional comparison from RCT	CFQ; Barratt Demographics Questionnaire	Child's weight and height; Child dietary intake	Lower levels of restrictive feeding were associated with lower child BMI-Z scores following intervention	17
Lee and Keller, 2012	Netherlands	4987 parents and children; 4	Cross-sectional	CEBQ; CFQ	Parent and child weight and height	Parents' Pressure to Eat was negatively related to child BMI	15
Jansen et al., 2012	USA	77 parents and children; 4.1(0.9)	Cross-sectional	CFQ	Parent and child weight and height; demographics	Child BMI was positively associated with parental concern about child weight and with lower perceived responsibility for feeding	21
Lee and Keller, 2012	USA	175 parents and children; 4–6	Cross-sectional	CFQ	Child's weight and height; test meals served in a lab	Pressure to eat was negatively associated with child BMI z score	15
Manan et al., 2012	Malaysia	175 mothers and children; 7–8	Cross-sectional	CFQ	Child weight and height; demographics	Pressure to eat among parents of overweight and obese children was significantly lower compared to healthy weight children. Food restriction was positively associated with child's BMI	16
Marshall et al., 2011	Australia	93 parents and children; 7.6(2.9)	Cross-sectional comparison from baseline measures from an RCT	General Nutrition Knowledge Questionnaire; CFQ; Family Food Environment questionnaire (FFEQ); CFPQ	Child and parent weight and height; demographics; 24-hour recalls for child's diet	Greater use of a combined guide-and-reward construct and higher levels of concern for their child's intake were significantly associated with lower child BMI-Z scores	15
Murashima et al., 2012	USA	330 mothers and children; 4.2(0.6)	Cross-sectional	Parental control over child feeding; the Block Food Screener (BFS);	Child and parent weight and height	Directive control predicted lower child BMI; Mothers' nondirective control was associated with intake of lower energy density and higher nutrient dense foods	20
Noor et al., 2012	Malaysia	204 parents and children; 9–12	Cross-sectional	CFQ (Malay version)	Child weight and height	Higher restriction associated with higher BMI; low SES associated with low BMI	15
Rollins et al., 2014	USA	180 mothers and daughters; 5.4(0.4)	Longitudinal	CFQ; Restricted Access Questionnaire (RAQ)	Child weight and height	Limiting or restricting snacks was associated with eating in the absence of hunger (EAH) at 5 years; Low inhibitory control girls had greater increases in EAH and BMI from 5 to 7 years	17
Taylor et al., 2011	Australia	175 parents and children; 9.2(1.1)	Cross-sectional	Authoritative Parenting Index (API); CFPQ; Parental activity-related practices; Child Nutrition Questionnaire (CNQ: preference for fruit and vegetable subscales); Children's Attraction to Physical Activity Questionnaire (CAPA)	Parent and child weight and height; parent-reported child diet and physical activity; child's preference for non-core foods; parents' level of education.	General parenting style was not associated with child BMI. Parental food restriction was associated with higher BMI whereas pressure to eat was associated with lower BMI	20
Tschann et al., 2013	USA	Parents of 174 Mexican American children (quantitative); *n* = 35 parents (qualitative); 8–10	Cross-sectional and qualitative	Parental Feeding Practices (PFP)	Parent and child weight and height; Sociodemographic information	Mothers and fathers differed in their use of feeding practices. Higher maternal positive involvement in feeding was associated with lower child BMI. Parental pressure to eat was associated with lower child BMI, parental restriction with higher child BMI. Fathers using food to control behavior had children with lower BMI	11
Webber et al., 2010a	UK	213 mothers and children; 7–9	Cross-sectional analysis of baseline data from longitudinal study	CEBQ; CFQ	Child weight and height; FMI; WC; demographics	Maternal restriction was associated with food responsiveness. Pressure to eat was associated with satiety responsiveness, slowness of eating, and fussiness. All effects were independent of child BMI	20
Webber et al., 2010b	UK	213 parents and children; 7–9	Cross-sectional analysis of baseline data from longitudinal study	CFQ	Child weight and height; FMI; WC; demographics; maternal perception of weight and concern about overweight	Higher BMI was associated with lower ‘pressure to eat’ and higher ‘restriction’ scores. Maternal concern about overweight mediated the association between child adiposity and restriction	20
Webber et al., 2010c	UK	213 parents and children; 7–9	Longitudinal	CFQ	Child weight and height; FMI; WC	No longitudinal association between child feeding practices at baseline and change in child adiposity. Higher child BMI at baseline was associated with smaller decrease in food monitoring and larger decrease in pressure to eat at follow-up	19
Wehrly et al., 2014	USA	243 parents and children; 4–6	Cross-sectional and cross-cultural comparison	CFQ	Child weight and height; demographics	Pressure to eat negatively associated with BMI. Restriction positively correlated with perceived weight	19
Zhang and McIntosh, 2011	USA	312 parents and children; 11.9(2.2)	Cross-sectional		Child weight and height; interviews about parental control over child feeding	Parental encouragement to eat enough was associated with a lower child BMI and helping children to eat a healthy diet was associated with a higher BMI	15

Abbreviations: BMI, Body Mass Index; CEBQ, Child Eating Behavior Questionnaire; CFQ, Child Feeding Questionnaire; CFSQ, Caregiver's Feeding Styles Questionnaire; CFPQ, Comprehensive Feeding Practices Questionnaire; FFQ, Food Frequency Questionnaire; FMI, Fat Mass Index; PDI(-S), Parenting Dimension Inventory(-Short Form); PFSQ, Parental Feeding Style Questionnaire; WC, Waist Circumference.

Papers were coded and scored (Table 1) for quality using an adapted rating scale (Moore, 2012). The scale contained 11 items such as: Is the study clear in what it seeks to do? (Is the purpose of the study discussed; Are the hypotheses presented; Are the outcomes to be measured clearly stated); Were the measures used valid and reliable? Each item was scored on a Likert scale using values of 0 = no; 1 = partly, and 2 = yes. The process resulted in each paper being given a total score to reflect quality.

The first author scored all of the papers and remaining three co-authors scored a random sample of 10 papers, resulting in 84% of the papers being scored for quality by at least two researchers. For papers with disagreements of more than three points on quality ratings, an additional independent researcher then scored the papers until an agreement was reached by re-reading the paper and extracting relevant data to support the scores. Scores which were off by 1–2 points were averaged. Finally, an inter-rater reliability coefficient was calculated. The findings indicated a high level of agreement (single measures interclass correlations by use of a one way random effects model, *r* = 0.85, *p* < 0.0001). Overall the papers included in this review exceeded a reasonable quality threshold, and most used measured BMI alongside validated measures of parenting/feeding style and/or feeding practices.

## Results

**Figure 2:**
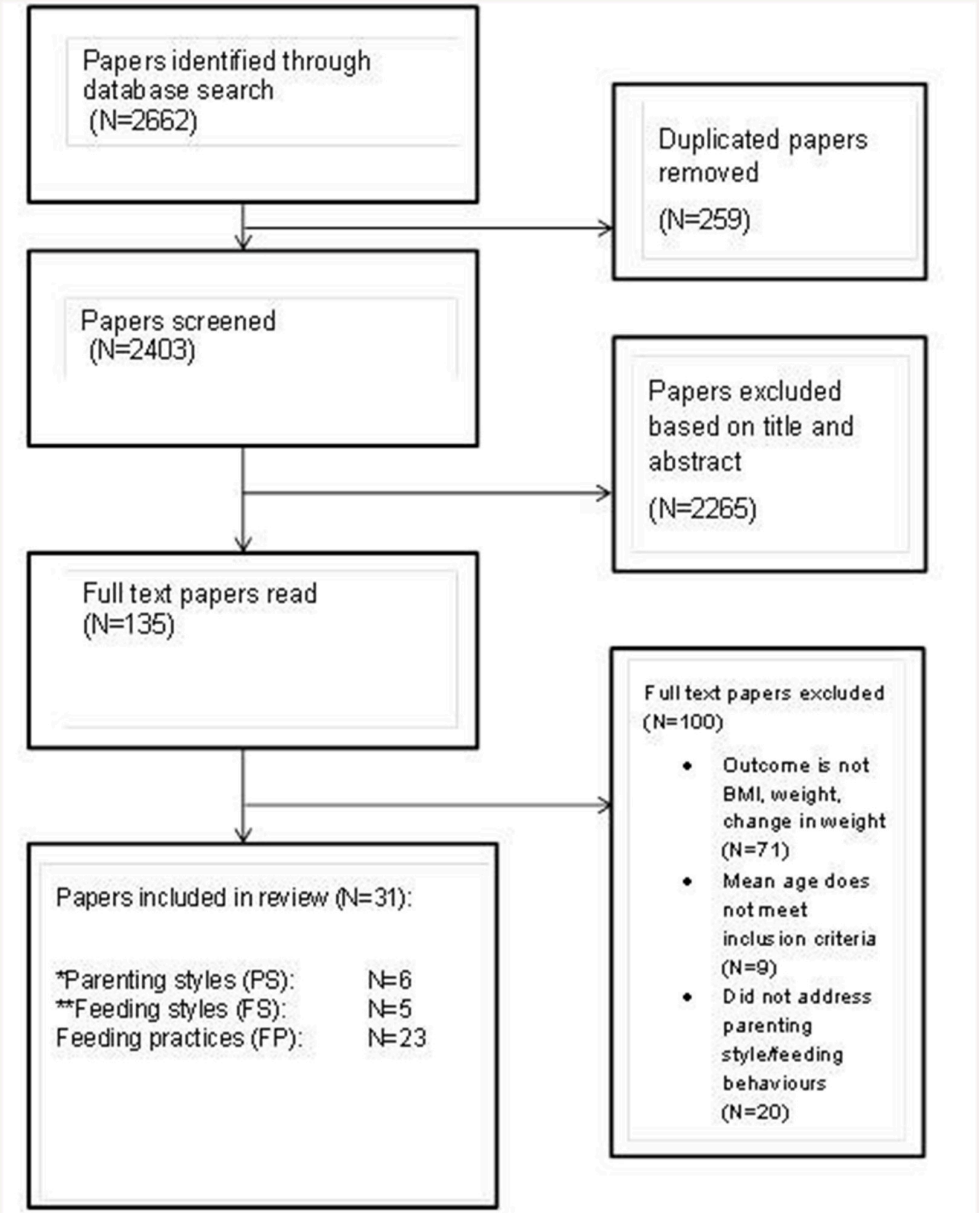
**Flow diagram study selection process for review**. ^*^Two articles addressed parenting style and feeding particles. ^**^One article addressed feeding style and parenting style.

The initial search identified 2662 papers. Two hundred and fifty-nine papers were removed due to duplication. Two thousand two hundred and sixty-five papers were excluded upon review of title and abstracts, mostly due to studies with participants outside the specified age range. This was expected as the word “child” was not used in our search to avoid missing research using alternative terms such as pre-adolescents, pre-schoolers, etc. Full text papers were reviewed for 135 studies of which 100 were excluded. These were excluded on the basis of the outcome measure, the age range or the absence of relevant behaviors (see Figure 2). The process resulted in 35 papers for inclusion in this review. Four papers were reviews (Thompson, 2010; Hurley et al., 2011; Sleddens et al., 2011; Vollmer and Mobley, 2013) and were therefore not addressed in the results section but only in the discussion. While most papers addressed the role of feeding practices, it was difficult at times to ascertain whether the authors had used the conventional definitions for each of the three domains of interest. For example, it has been reported by Blissett (2011) that inconsistencies in the use of these definitions is problematic, with terms such as feeding style confused with feeding practice, so there are times when terms are not clearly conceptualized or they are used synonymously. Therefore, within this review the authors' use of the construct has been applied but where definitions overlap or are used interchangeably, this is noted. Due to the variety of measures used and potential lack of precision, the effect sizes are not reported in the current review.

### Overall study characteristics

The papers identified by the review process produced a range of sample sizes including relatively small (*n* = 69, Olvera and Power, 2010) to large scale studies like that of Jansen et al. (2012) with 4987 children. The age range covered 2–18 years within the papers, but the focus of this review was 4–12 years representing school age and the age most likely to be influenced by parenting and the home environment. Studies focused on mostly mothers and their interactions with their children with only one study addressing the differences between maternal and paternal feeding practices (Tschann et al., 2013). Most were conducted in the USA (*n* = 15), others were conducted in Australia (4), the UK (4; one of which had a German comparison group), the Netherlands (4), Malaysia (2), Brazil (1), and Turkey (1). Studies were ethnically and economically diverse, but few were sufficiently powered to assess the specific contribution of ethnic differences to the association between parenting and child BMI. All but one of the studies included both boys and girls; the exception was Rollins et al. (2014) with a girl-only sample. Only one study addressed both parents' and children's perceptions of parenting styles (Taylor et al., 2011). The main characteristics of the papers revealed by the review process such as sample size, country of origin, age range, design, measurements used, and main findings are presented in Table 1.

### Parenting styles

#### Characteristics of the studies

The search revealed six papers addressing parenting styles. Three were longitudinal studies (Olvera and Power, 2010; Rodenburg et al., 2012; Hancock et al., 2014); and three were cross-sectional (Hennessy et al., 2010; Rodenburg et al., 2011; Taylor et al., 2011).

#### Instruments used

Of the six studies investigating parenting style, four different instruments were used: the Parenting Dimensions Inventory was used in two studies (Hennessy et al., 2010; Olvera and Power, 2010), the Authoritative Parenting Index was used by one (Taylor et al., 2011), a Dutch translation of a parenting style measure was used by the same group in two papers (Rodenburg et al., 2011, 2012), and the protective parenting measure was applied in one study (Hancock et al., 2014).

#### Findings

In all three longitudinal studies identified since 2010, parenting style was significantly associated with child weight. Olvera and Power (2010) investigated the change in weight from baseline to follow-up (3 years later) in Mexican-American children and their mothers. These authors found that when controlling for initial weight status, children whose mothers followed an indulgent or uninvolved parenting style at baseline were more likely to become overweight than children of authoritarian or authoritative mothers. In the study by Rodenburg et al. (2012) the potential interaction effect between parenting style and child appetite traits were examined from baseline to 1 year follow-up. In this study, food approach traits such as food responsiveness and enjoyment of food predicted a higher child BMI z-score and all food avoidance traits predicted a lower child BMI. An uninvolved (“neglectful”) parenting style amplified the relationship between food approach traits and a higher BMI z-score. This illustrates the importance of considering child characteristics as well as parenting styles when assessing obesity risk. Within the Australian longitudinal cohort where protective parenting was used to predict BMI over a period of 5 years (data collected three times), high maternal protectiveness measured at 4–5 years old was not significantly associated with weight until children reached the ages of 10–11 years (Hancock et al., 2014). By this time, protectiveness was associated with a higher odds ratio of the child being overweight or obese. This suggests that the effects of some parenting styles may emerge later in childhood.

Among the three cross-sectional studies, parenting style was a significant predictor of child BMI in only one, large scale study of 9 year-old children (*n* = 1275; Rodenburg et al., 2011) and failed to reach significance in the other two smaller studies (Hennessy et al., 2010; Taylor et al., 2011). Child BMI z-score was associated with a more rejecting parenting style which is described as low support, low behavioral control but high psychological control (Rodenburg et al., 2011). The study by Hennessy et al. (2010) of 91 families with 9 year-old children reported a modest association between parenting style and feeding style; but only feeding style was linked to child BMI z-score (see next section). Although the study by Taylor et al. (2011) of 7–11 year old children (*n* = 175) found no association between parent-reported parenting styles and child BMI, child-perceived parenting style was significantly associated with diet and activity. In this case, when children perceived parents to be more demanding and responsive, which could be considered a proxy for authoritative parenting, they showed healthier weight-related attitudes such as liking for fruit and vegetables and appreciation of physical activity. The potential for a moderating role of the child's perception of parenting style is worthy of further consideration.

Overall, only the longitudinal studies showed that parenting style was consistently associated with child BMI; the cross-sectional studies were more mixed in their findings, perhaps attributable to smaller sample sizes. It can be concluded from the stronger evidence extracted from longitudinal studies that parenting style is associated with child BMI over time and these effects may be influenced by child age and eating traits.

### Feeding styles

#### Characteristics of the studies

In total, five papers investigated feeding style and its association with child BMI and they all used a cross-sectional design (Hennessy et al., 2010; Hughes et al., 2011; Tovar et al., 2012; Yilmaz et al., 2013; Frankel et al., 2014).

#### Instruments used

Most studies (Hennessy et al., 2010; Hughes et al., 2011; Tovar et al., 2012; Frankel et al., 2014)measured feeding style using the Caregiver Feeding Style Questionnaire (CFSQ; Hughes et al., 2005. One study (Hughes et al., 2011) cross-validated its findings from the CFSQ with findings from observational measurements made at three dinner meals, using the Feeding Behavior Coding System (FBCS; Hughes et al., 2007), an observational checklist that documents specific feeding practices and their frequency of occurrence. This study had a high quality rating as it allowed comparison of parents' self-reported data with observed behaviors. Yilmaz et al. (2013) used the Parental Feeding Style Questionnaire (Wardle et al., 2002) which measures instrumental feeding, emotional feeding, encouragement to eat and control over eating. In addition, the validated Turkish version of the PFSQ divided the control scale into two subscales: strict control (parent decides when/what/how much child should eat) and permissive control (allowing child to make these decisions).

#### Findings

In three of the five cross-sectional studies, indulgent feeding styles were associated with higher weight or BMI z-scores (Hennessy et al., 2010; Tovar et al., 2012; Frankel et al., 2014). Where both parenting styles and feeding styles were evaluated in the same families it was found that the indulgent *feeding* style was associated with child BMI, but not *parenting style* (Hennessy et al., 2010).

Mothers of Turkish 5–7 year-olds who perceived their child to be overweight reported less use of emotional feeding styles or encouragement to eat on the Parental Feeding Style Questionnaire (PFSQ; Wardle et al., 2002; Özçetin et al., 2010; Yilmaz et al., 2013). This suggests that parental perception of child's weight may influence their use of feeding styles or practices.

Within a sample of African American and Hispanic American families, an indulgent feeding style was associated with lower satiety responsiveness, higher enjoyment of food, and a higher BMI in the children (Frankel et al., 2014). The study by Hughes et al. (2011) found that Hispanic boys with indulgent parents had significantly higher BMI z-scores compared to Hispanic boys in the other three feeding style groups (authoritative, authoritarian, and uninvolved), but this association was not seen in Hispanic girls or in African American children. The importance of gender and ethnic differences in determining the relationship between feeding style and child BMI are evident. Tovar et al. (2012) studied feeding styles among recent immigrants to the USA. They found that among Haitian, Brazilian, and Latina women, authoritarian and indulgent feeding styles were the most common. Similar to findings from studies in non-immigrant populations, the indulgent feeding style was positively associated with a high child BMI z-score across this diverse sample.

Overall, these findings illustrate the more consistent relationship between feeding style and risk of obesity compared to more general measures of parenting style.

### Feeding practices

#### Characteristics of the studies

Most of the papers identified in this review investigated feeding practices. Seventeen studies were cross-sectional (Hennessy et al., 2010; Webber et al., 2010a,b; Costa et al., 2011; Marshall et al., 2011; Taylor et al., 2011; Zhang and McIntosh, 2011; Cardel et al., 2012; Jansen et al., 2012; Lee and Keller, 2012; Murashima et al., 2012; Manan et al., 2012; Noor et al., 2012; Blissett and Bennett, 2013; Dev et al., 2013; Tschann et al., 2013; Wehrly et al., 2014); four were longitudinal (Campbell et al., 2010; Webber et al., 2010c; Gubbels et al., 2011; Rollins et al., 2014); and one was a randomized control trial (Karp et al., 2014).

#### Instruments used

The Child Feeding Questionnaire (CFQ) developed by Birch et al. (2001) was the most commonly used measure to assess feeding practices and was used in 18 of the 22 empirical studies (Campbell et al., 2010; Hennessy et al., 2010; Webber et al., 2010a,b,c; Costa et al., 2011; Gubbels et al., 2011; Marshall et al., 2011; Cardel et al., 2012; Jansen et al., 2012; Lee and Keller, 2012; Manan et al., 2012; Noor et al., 2012; Dev et al., 2013; Holland et al., 2014; Karp et al., 2014; Rollins et al., 2014; Wehrly et al., 2014).

In one study (Marshall et al., 2011) the CFQ was used as part of a larger parenting questionnaire which also included the Comprehensive Feeding Practices Questionnaires (CFPQ; Musher-Eizenman and Holub, 2007) and the Family Food Environment questionnaire (Campbell et al., 2006). Both studies by Blissett and Bennett (2013) and Taylor et al. (2011) used the CFPQ whereas Tschann et al. (2013) used the Parental Feeding Practices questionnaire, which was adapted from items taken from several validated parental feeding practice measures such as the CFPQ and CFQ. In each case, the measures included scores for monitoring, restriction and pressuring to eat.

Murashima et al. (2012) used a questionnaire they had developed (Parental Control over Child Feeding; Murashima et al., 2011) assessing different types of control applied to child feeding situations. The study by Zhang and McIntosh (2011) assessed feeding practices such as encouraging adequate intake, keeping track of types of foods eaten and building healthy habits using telephone-based interviews. One study also asked children to report on the extent to which their mother controlled access to snack foods (Rollins et al., 2014). Hughes et al. (2011) complemented self-report measures from the CFSQ by observational measures as mentioned above.

### Restriction/control feeding

Of the original research studies, 14 observed a significant association between restrictive/controlling feeding and high child BMI, although this was more strongly evidenced within the cross-sectional studies (see Table 2). Within these studies, one study found the association was specific to girls with low inhibitory control (Rollins et al., 2014) and one study had mixed findings showing positive associations in some subgroups based on ethnicity (Blissett and Bennett, 2013). One study showed a negative association between restriction and BMI in younger, but not older, children (Campbell et al., 2010). Four studies reported no associations with weight (see Table 2).

**Table 2:** **The associations between parenting styles, feeding styles and feeding practices with child's weight status**.

**Year and author**	**Parenting style (P)/Feeding style (F)**					**Feeding practices**			
	**Indulgent**	**Authoritative**	**Authoritarian**	**Uninvolved**	**Other**	**Restriction**	**Pressure**	**Monitoring**	**Other**
Campbell et al., 2010						−^ˆ^			
Hennessy et al., 2010	P0	P0	P0	P0		−	−	0	
Olvera and Power, 2010	P+	P−	P−	P0					
Webber et al., 2010a						0	0	0	
Webber et al., 2010b						0		0	
Webber et al., 2010c						0		0	
Costa et al., 2011						+	0	0	
Gubbels et al., 2011						+	−	−	
Hughes et al., 2011	F+^#^	F0	F0	F0					
Marshall et al., 2011								0	^a^
Rodenburg et al., 2011		P−		P+					
Taylor et al., 2011					^b^	+	−	0	
Zhang and McIntosh, 2011						0		0	
Cardel et al., 2012						+	−		
Jansen et al., 2012						+	−	0	
Lee and Keller, 2012							−		
Manan et al., 2012						+	−	0	
Murashima et al., 2012						+			
Noor et al., 2012						+	−	0	
Rodenburg et al., 2012	P0	P0	P0	P0					
Tovar et al., 2012	F+								
Blissett and Bennett, 2013						+	0	0	
Dev et al., 2013						+	0	0	
Tschann et al., 2013						+	−	−	
Yilmaz et al., 2013					^c^				
Frankel et al., 2014	F+			F+					
Hancock et al., 2014					^d^				
Holland et al., 2014						+	−	−	
Karp et al., 2014						+	−	0	
Rollins et al., 2014						+^%^			
Wehrly et al., 2014						+	−		

*0, No association/differences found*.

*+, positive association with child BMI*.

*−, inverse association with child BMI; grayed out cell indicates that association was not tested*.

ˆ *Higher baseline restriction scores were associated with lower BMI z-scores at 3 year follow up in 5–6 year-old, but not in 10–12 year-old, children*.

# *Positive association only in Hispanic boys, but not other groups*.

% *In children with low inhibitory control*.

a *Rewarding feeding styles was associated with lower BMI*.

b *Responsive and demanding parenting style dimensions were not associated with child BMI*.

c *Emotional feeding and encouragement to eat were lower in children perceived to be overweight*.

d *Greater maternal protectiveness at age 8–9 (but not at 6–7) was associated with children being overweight or obese at ages 10–11*.

The four longitudinal studies were equivocal about whether restriction and controlling feeding practices were associated with obesity risk. Two studies found no longitudinal associations between restriction and change in BMI (Webber et al., 2010c; Gubbels et al., 2011). In the study by Campbell et al. (2010) baseline restrictive feeding was not associated with baseline BMI in younger (5–6 years) or older (10–12 years) children. However, when the younger children were measured at the 3 year follow-up, parents who were more restrictive at baseline had children who gained less weight over time. It was suggested that restriction can have *protective* effects against unhealthy weight gain in younger, but not older, children (Campbell et al., 2010). This supports the idea that some feeding practices such as setting limits in early life may reduce obesity risk but this practice has less of an effect on older children.

Taking child appetite characteristics into account, the longitudinal study by Rollins et al. (2014) examined controlling feeding practices including restricting access to snacks and change in weight over 3 years. In this study four feeding profiles were identified according to the type of limit-setting or restriction reported by mothers: (1) unlimited access to snacks; (2) sets limits and does not restrict snacks; (3) sets limits and restricts high fat/sugar snacks; (4) sets limits and restricts all snacks. The girls whose mothers restricted all snacks showed higher levels of eating in the absence of hunger (EAH) at 5 years. The girls with low inhibitory control and high levels of approach (strong temperamental anticipation of expected reward) were heavier than those with high control and low approach. In addition, girls with low inhibitory control whose mothers provided unlimited access to snacks or restricted all snacks gained the most weight and increased EAH between 5 and 7 years. This study is important in illustrating that characteristics of the child are essential in understanding the links between child BMI and feeding practices; also the study demonstrates the nuanced relationship between restriction and child BMI. On the one hand, restricting all snacks was associated with EAH, and typically this behavior increases the risk of later obesity. On the other hand, both extremes of limit setting (restricts all snacks or free access to snacks) did not produce the desired moderating effect on BMI in girls with low inhibitory control.

Of the cross-sectional studies, 12 found that restrictive feeding was linked to child overweight and obesity (see Table 2). Thus, the higher the child BMI, the greater the reported level of restrictive feeding practice. Higher levels of restrictive feeding have also been associated with maternal perceptions that the child has a large appetite (Webber et al., 2010a) or concerns about child overweight (Webber et al., 2010b), which may mediate the association between child adiposity and restriction (Webber et al., 2010b). This suggests that parents may use restriction in response to the child's perceived weight status or appetite.

Maternal concern about child overweight was also associated with a linear increase in restriction across weight groups, and this maternal concern about overweight mediated the association between child adiposity and restriction (Webber et al., 2010b), suggesting that parents use restriction in response to child's perceived weight status. In another study with a large cohort of children, Jansen et al. (2012) reported significant cross-sectional associations between restrictive feeding and child weight. Noor et al. (2012), in a study of Malay children, also found that parents of overweight children controlled their child's intake by restricting amount of food given. This suggests that restriction is applied to heavier children across different contexts and cultures.

Where different groups have been studied within a single study, it is also interesting to note that restrictive practices may differ by sub-group. For example, Cardel et al. (2012) reported significantly higher levels of restrictive feeding in Hispanic American parents compared to European American or African American parents. In another study, higher restrictive feeding practices were associated with higher child weight, only for British Black Afro Caribbean (BAC) but not for white British (WB) or German (WG) families (Blissett and Bennett, 2013). In this instance, restrictive feeding practices were also linked with child eating traits. BAC parents reported that their children showed the highest food approach behaviors; in turn, higher food approach was associated with higher child BMI in both BAC and WG groups. Parents may therefore apply restriction as a means to control eating behavior in their children with high food approach tendencies.

Wehrly et al. (2014) examined two distinct dimensions of control in an ethnically and economically diverse group of American children: restriction of unhealthy foods and pressure to eat healthy foods. They found these feeding practices were positively correlated with each other and each was independently associated with child BMI and body fatness. Although it seems surprising that restriction and pressure are positively correlated when typically they are inversely correlated, this can be explained by pressure or encouragement to consume healthy foods while limiting or restricting access to unhealthy foods in children who are overweight. Thus, the correspondence between the two relates to different ways to obtain the same outcome, namely healthy eating. In addition, they found that parental perception of child overweight was linked to the use of restriction. These findings confirm those by Webber et al. (2010b) that parental perception of child weight status is an important determinant of feeding practices.

Feeding practices such as restriction are subject to parental perception of their child's weight and are modifiable. This was demonstrated in the trial by Holland et al. (2014) who conducted a 16-session family-based behavioral weight loss treatment with families of 7–11 year old children. Across the time of the trial, restrictive feeding practices decreased at the same time as weight loss. During the period of the intervention, as children lost weight, and reduced their total energy intake, parents reported a decrease in their concern about their child's weight and an increase in perceived responsibility for quantity and quality of the child's diet. Children decreased the percentage of total energy intake from fat and increased energy from protein. They reduced intake of sugars and increased intake of fruits and vegetables. Mediational analyses found a significant mediating effect of selective dietary changes between the change in restrictive feeding and change in child BMI. This suggests that selective restriction (high fat foods, total energy intake) helped to promote a healthier BMI in this study. The trial had no control group, therefore caution must be applied to the results of this study. But the suggestion is made that limit setting, rather than excessive restriction, was effective in weight loss for these children.

Across the four longitudinal studies, restrictive feeding practices were not consistently related to weight change over time. One found weight gain was more related to child inhibitory control than restriction (Rollins et al., 2014) and one showed a protective effect of restriction applied to younger children. Within cross-sectional studies, a consistent finding across studies and cultures was that restriction was associated with a high child BMI, suggesting the possibility of reverse causality. In other words, the restrictions imposed by parents could be a response to child eating traits and weight status, rather than a causal factor in developing overweight, although this was not found in the one study that explored this possible relationship longitudinally (Webber et al., 2010c).

### Pressure to eat

Pressure to eat is a practice parents may use to encourage or cajole their child to eat enough food. Pressure to eat was associated with lower child BMI in 11 cross-sectional studies (Hennessy et al., 2010; Webber et al., 2010b; Costa et al., 2011; Taylor et al., 2011; Cardel et al., 2012; Jansen et al., 2012; Lee and Keller, 2012; Manan et al., 2012; Noor et al., 2012; Tschann et al., 2013; Wehrly et al., 2014), one longitudinal (Gubbels et al., 2011), and one part of a RCT (Karp et al., 2014). No association was found in another of the longitudinal studies (Webber et al., 2010c). In the longitudinal study which did find an effect (Gubbels et al., 2011) the authors adapted the pressure to eat scale to specifically investigate “stimulation of healthy intake” and found that this variable was significantly concurrently associated with higher child BMI z-score at age 5 and predicted a healthy BMI at age 7. Therefore, parents encouraged heavier children to accept more healthy foods in their diets at age 5 and this improved BMI by age 7 (Gubbels et al., 2011).

Although there were studies finding no associations between parental pressure to eat and child weight (Blissett and Bennett, 2013; Holland et al., 2014), findings from the remaining seven studies generally suggested that pressure to eat was associated with a low child BMI. This pattern was also seen in non-Western cultures with two studies of children in Malaysia finding that parents of heavier children were less likely to pressure their child to eat than parents of children with a normal (Manan et al., 2012) or low BMI (Noor et al., 2012).

In another study involving an ethnically and economically diverse sample, pressure to eat differed by parental ethnicity and by family income (Wehrly et al., 2014). As such, White Non-Hispanic parents reported lower pressure to eat compared to White Hispanic, Black, and Asian parents. In addition, household monthly income was inversely related to pressure to eat, parents' perceptions of child weight status, and child BMI. Similarly, while Blissett and Bennett (2013) reported ethnic differences in feeding practices, such that White German parents reported lower levels of pressure to eat compared to White British and Black Afro-Caribbean mothers, this was not linked to weight. Pressure to eat might also vary by parental gender, with one study (Tschann et al., 2013) finding that fathers reported higher levels of pressure to eat compared to mothers.

Webber et al. (2010a) reported that characteristics of the child's appetite were associated cross-sectionally with feeding practices applied by parents. Thus, children who were described as fussy or as slow eaters had parents who reported higher levels of pressure to eat, and those with high enjoyment of eating had lower pressure to eat. But these effects were independent of child BMI. Exploring the possibility that parents reacted to child characteristics longitudinally, it was found that higher baseline BMI predicted a greater decrease in pressure to eat over time (Webber et al., 2010c).

### Monitoring child intake

Among 17 papers investigating the associations between monitoring and weight, most studies (*n* = 14) found no link between monitoring intake and child BMI (see Table 2). In fact, some forms of monitoring child intake may protect against obesity.

Of two longitudinal studies, one found no relationship between monitoring and child weight (Webber et al., 2010c), but the other found that parental monitoring of child intake at 5 years of age was associated with lower weight and better diet quality at age 7 (Gubbels et al., 2011).

One study examined a wide variety of feeding practices as part of a larger RCT to identify the role of learning and co-participation in eating decisions on child BMI (Marshall et al., 2011). The authors explored different factors emerging from an exploratory factor analysis and included a factor on tracking and talking about food which includes monitoring intake of high fat foods, sweet, and savory snacks, discussing the importance of health, the nutritional value of foods, and encouraging intake of healthy foods before unhealthy foods. Another factor, named “guide and reward” included parental efforts to regulate their child's intake such as guiding intake away from favorite or “junk” foods; ensuring that their child does not eat too many sweets and offering favored foods as rewards for good behavior. This factor with a monitoring item was significantly associated with high child BMI, but because this factor included so many variables it is difficult to draw specific conclusions about monitoring. Concern about poor intake was inversely related to child BMI.

Ethnic differences in parental feeding practices were found by Blissett and Bennett (2013). In this study Black Afro Caribbean parents reported the lowest levels of monitoring (and highest restriction) compared to White British and German parents.

Exploring potential bi-directional relationships, a longitudinal study reported that a high baseline child BMI was associated with a lower decrease in the use of monitoring over time (Webber et al., 2010c). This maintenance of monitoring levels in response to a high child BMI could be related to the child's tendency to overeat (Webber et al., 2010a). It has been argued that monitoring might be protective against overweight (Gubbels et al., 2011), and that this response by parents can be beneficial. If parents are concerned about child overweight and overeating, then monitoring is an obvious surveillance strategy.

### Positive involvement

The concept of “positive involvement” encompasses several “protective elements,” defined by Tschann et al. (2013) as parents limiting foods high in energy content, providing small, appropriate servings of food, and asking children about what they ate. This construct incorporates restriction of specific foods, limiting portion size and monitoring intake. Parental positive involvement was significantly correlated with pressure to eat and restriction of amount of food. Maternal (but not paternal) positive involvement was associated with a lower child BMI (Tschann et al., 2013). These results confirm that some aspects of restriction and monitoring may be considered protective against overweight and supports the proposal that structure and support may benefit children; however, it is not known which of the elements within positive involvement are responsible for the protective effects.

## Discussion

The primary objective of the current review was to identify modifiable parental behaviors associated with overweight and obesity in children aged 4–12 years. Overall in this age group, the evidence suggests that the indulgent and uninvolved parenting and feeding styles were associated with a higher child BMI. Associations between parenting style and child BMI were strongest and most consistent within the longitudinal studies; whereas for specific feeding practices such as restriction and pressure to eat, these were linked to BMI most strongly within cross-sectional studies. Measures of feeding style were more consistently related to the risk of obesity than were more general measures of parenting style. Few studies identified protective feeding practices, but in one case, monitoring was linked with lower BMI in a longitudinal context (Gubbels et al., 2011) and positive involvement in one cross-sectional study (Tschann et al., 2013). In addition, studies highlighted the importance of child characteristics such as inhibitory control or eating traits, ethnicity, age, and socioeconomic status as moderating factors in the relationship with child BMI.

### Parenting styles

Indulgent or uninvolved parenting was generally linked to higher child BMI (e.g., Hughes et al., 2008; Olvera and Power, 2010). These findings are consistent with previous literature as these groups were evaluated in comparison to the authoritative style. In support, Sleddens et al.'s (2011) review of the literature notes that authoritative parenting produced healthier children (healthy eating, active lifestyles, and lower child BMI); whilst indulgent feeding was associated with more negative health outcomes. This was also consistent with the review by Vollmer and Mobley (2013), who also found the authoritative style to be protective against obesity. It should be noted that the literature in favor of the authoritative parenting style has been so consistent, across a number of child outcomes, that many of the recent papers on parenting/feeding style used this style as the “reference” group. Whereas, an authoritative parenting style combines expectations about adherence to a healthy diet and sets limits on certain foods, an indulgent style sets few expectations and gives children greater freedom to choose what is eaten. Previous research has also linked authoritative parenting to healthy diets (e.g., Kremers et al., 2003; De Bourdeaudhuij et al., 2008). A recent review by Collins et al. (2014) explored the relationship between parenting styles and its associations with feeding practices. Although in this review, child weight was not a primary outcome, the authors suggested that an authoritative parenting style was associated with parental monitoring of child food intake (which has been previously associated with healthy eating, and thus lower child BMI).

Given the importance of these observations for promoting authoritative parenting, there is a clear need to use consistent measures of parenting styles across studies. In the present review, four different measurement tools were identified across the six studies to investigate parenting style and a wide range of definitions was applied. Consensus in definition, concepts and measurements will lead to greater consensus in study outcomes, as suggested by Blissett (2011).

### Feeding styles

Mixed findings were identified for feeding styles. Authoritative feeding styles were protective against the risk of obesity and were associated with a higher consumption of fruit and vegetables (Rodenburg et al., 2012) but not weight. Authoritative feeding styles with clear rules and boundaries promoted a healthy BMI (Rodenburg et al., 2012). However, only cross-sectional studies were found in this search, and this limits the conclusions which can be drawn about feeding styles over time. Similar to parenting styles, indulgent feeding styles were also associated with risk of overweight and obesity, a finding which was consistent with previous reviews (Hurley et al., 2011; Vollmer and Mobley, 2013).

Evidence of ethnic differences and their associations with different feeding styles was reported. As such, Tovar et al. (2012) noted that feeding styles significantly differed by child BMI z-score and by ethnic group. The association between feeding style and child weight was also found to vary between children of different genders or ethnicity (e.g., Hughes et al., 2011). Therefore, many family factors such as culture, ethnic group, gender, or SES might moderate the effect of feeding styles on child weight.

### Feeding practices

Specific feeding practices were linked to child BMI across a number of studies, supporting existing observations of these relationships (e.g., Faith et al., 2004). Among the studies in the current review, restrictive/controlling feeding practices were generally linked to higher child BMI, whereas pressure to eat was associated with lower child BMI. Interestingly, restriction and pressure to eat were positively correlated with one another when restriction was specifically applied to unhealthy foods and pressure to eat on healthy foods (Wehrly et al., 2014). In this case, both behaviors were directed at children with a high BMI, and may have been two practices used to guide children toward the goal of healthier eating. Whether this is effective is not yet evident. What is clear is that within each of these constructs, the distinction must be made between negative and positive consequences. Excessive restriction or strict limit setting can be linked to parental efforts to prevent overeating compared with more positive and effective limits placed on intake (such as appropriate portion sizes) which serve to guide healthy food intake. Similarly, pressuring children to eat when they are not hungry (potentially reducing the child's ability to self-regulate intake) should be considered distinct from positive encouragement to eat certain healthy foods as part of guiding choice and providing appropriate “structure” (Grolnick and Pomerantz, 2009).

Hurley et al. (2011) reviewed the literature regarding two practices related to responsive feeding finding positive associations between nonresponsive feeding and child weight in 24/31 papers. The integrative review by Thompson (2010) identified 18 papers addressing feeding practices and childhood obesity in pre-school children. In this review, it was found that culture, parental education and parents' own eating behaviors were all significantly associated with child weight. In particular, risk of obesity was associated with how parents *controlled and modified their children's eating*, confirming the relationship between pressure to eat and low child BMI and between restriction and high child BMI.

Cross-sectional studies provide a snapshot but fail to take account of how parents respond to their children over time and how this evolves as children grow. The cause and effect direction is not known. It is feasible that restriction is applied to children with high food approach tendencies (e.g., Webber et al., 2010b). Similarly, parents may pressure children to eat who are fussy or who seem to be undereating (Webber et al., 2010b). Encouragement to eat a healthy diet and monitoring intake to ensure children are eating well could be protective against obesity, and may be applied more generally by parents, regardless of the child's weight status. This was illustrated by Gubbels et al. (2011) where monitoring children's intake and stimulating healthy food choices at age 5 years predicted a healthier child BMI at age 7 years.

A few large-scale longitudinal studies have explored the reciprocal relationship between feeding practices and weight. The analyses by Webber et al. (2010c), revealed evidence of a child responsive model of parenting. Pressure to eat was applied *in response* to children who had a lower BMI and monitoring was maintained at a higher level for children with higher BMI. The argument for a child responsive model in determining feeding practices is persuasive. It may explain the consistent finding of associations between child weight status and parents' use of specific feeding practices. Parents may respond differently to individual children within their own family, taking child characteristics, and eating behaviors into account. Jansen et al. (2014) further explored the directionality of the relationship between feeding practices (using the CFQ at age 4 years) and child BMI longitudinally (ages 2, 4, and 6 years). They demonstrated that child BMI at 2 years of age predicted parents' use of restriction and pressure to eat (but not monitoring) at age 4. Such behaviors also predicted changes in BMI at 6 years old. Although the associations were reciprocal, a stronger association was observed for parental response to the child's weight.

Gerards and Kremers (2015) identified a link between parental behavior (parenting styles and food parenting practices) and children's weight-related outcomes. They suggest that general parenting style may moderate the impact of feeding practices on children's nutrition behaviors. Feeding practices should therefore be considered in the context of both what the child eats (content of the meal), the atmosphere provided by parents, and the eating traits expressed by children. Early work on this topic is beginning to emerge, with studies exploring how caregivers both react to and influence children's behavior (Tovar et al., 2015).

It is also important to account for moderators such as child age, parent gender, family income, ethnic group, and family eating behaviors (e.g., parental involvement in child's eating, e.g., Tschann et al., 2013), when addressing feeding practices. Gender of the parent might moderate the effect of feeding practices on child's weight and ethnic groups may reveal differences in feeding practices (Blissett and Bennett, 2013). There are several possible reasons for these reported differences. It is possible that the higher frequency of use of behaviors, larger variation in reported behavior, or simply sample size differences between groups might result in different statistical outcomes. On the other hand, it is also possible that parents from different cultural backgrounds may use similar parenting practices to achieve different goals: some parents may use restriction for weight control or health, whereas others may aim to limit the amount of food consumed so that foods will last longer when budgets are constrained. These differences could therefore reflect differences in affluence rather than culture (Tovar et al., 2013). Future cross-cultural research could explore these motivational factors in greater detail.

In summary, specific feeding practices are linked to the risk of overweight and obesity. In particular, restricting food intake has been associated with higher child BMI. As previously mentioned, these practices may be used in response to the child's behavior. In either case, the use of certain feeding practices might be subject to additional considerations such as ethnicity, socioeconomic status, age, and gender. Thus, it is possible that the ways in which researchers refer to these common feeding practices mask the complexity of the parent-child interaction and the various strategies parents may use with their children to influence eating behavior (Tovar et al., 2013). This complex interaction between parent and child traits may explain why the use of feeding practices may differ even between siblings living in the same household (Saelens et al., 2000; Farrow et al., 2009).

### Limitations

The search applied in this review only identified peer-reviewed papers published in English. It is possible that relevant studies in other languages, as part of a thesis, or in journals not indexed in the databases searched may not have been identified.

A limitation of the present review was the lack of studies which investigated feeding practices such as modeling, use of rewards, instrumental, and emotional feeding (see Gevers et al., 2014 for a more complete list). A possible explanation for this is the rigid criteria applied for the review which excluded papers where body weight of the child was not one of the main outcomes of the study. Therefore, a range of studies have investigated different feeding practices, but if measured BMI was not provided then these were not included here.

A significant limitation identified in this review is the lack of consensus among researchers, firstly in applying the definitions of the constructs and in identifying “gold standard” measures for assessing parenting and feeding behaviors. Parenting styles, feeding styles, and feeding practices were conceptualized in the literature via a range of definitions and thus the ability to draw conclusions across such diverse ways of measuring each element as it links to child weight is challenging. Blissett (2011) reports that the literature has been clouded by the various ways concepts are defined or applied, it is therefore important to ensure that standard, agreed definitions are used in this field. Similarly, Vaughn et al. (2013) identified 71 instruments to measure feeding practices, with some having better reliability and validity than others. The authors highlight that although many measures are similar in content, they vary enough to make it difficult to compare results across studies. For this reason, a reliable, validated “gold standard” measure for assessing feeding practices would facilitate future research in this field.

Since findings from the studies in this review are based almost entirely on parent-report, there is a risk of biased or inaccurate outcomes, with parents potentially misreporting their behaviors due to lack of awareness, social desirability bias, or differences in use of rather subjective scales (e.g., differences in interpretation of how much is “often”). Further, a recent review by Bergmeier et al. (2015) found that across several observational studies, parents' reports of their feeding practices on a questionnaire were not correlated with the frequency of practices observed. The most commonly used questionnaire assessing feeding practices in the studies reviewed was the CFQ. Although this questionnaire has been previously validated, it addresses parental perceptions of how they feed their children and does not provide direct measures of these behaviors. Some of the questions may assess parents' attitudes more than their actual behaviors. For example, items such as “If I did not guide or regulate my child's eating, she would eat too much of her favorite foods” or “I have to be especially careful to make sure my child eats enough” are perhaps measuring parents' concern about child weight more than the frequency with which parents are using a particular feeding practice (restriction or pressure to eat, respectively, in the examples above). This may explain why scores on these scales are so well correlated with child weight status, but may not accurately reflect the parents' mealtime behavior, and therefore the actual association between feeding practices and child weight. A heavy reliance on self-report measures may underpin cross-cultural differences in studies, due to potential differences in participant response style. In such circumstances, observational studies would be particularly valuable.

Since many of the studies were cross-sectional, the ability to attribute causality to the findings is limited Gerards and Kremers, 2015. Since a child's development is shaped by the reciprocal interactions between parent and child (Sleddens et al., 2011; Skouteris et al., 2012), recognizing that parents may be responding to child attributes could be valuable in designing bi-directional longitudinal studies.

Another limitation is the wide range of ages we addressed (4–12 years). It is possible that some feeding practices may have a larger impact at particular periods of child development, whereas they may have different effects at other time points. For example, young children may benefit from restriction (Campbell et al., 2010) and an authoritative parenting (Vereecken et al., 2004) simply as they require more guidance, whereas as the child ages, such practices may cease to be beneficial as the child's autonomy increases (Savage et al., 2007). Furthermore, little is known about the stability of feeding styles and practices over time. Parenting and feeding styles measured within a cross-sectional study provide some insight into a limited period, but this cannot be assumed to be static. It is likely that parents adjust their approach to feeding as children develop and as they adopt different eating habits through experience within and beyond the family setting. This provides further support for the need for more longitudinal studies in this area.

Finally, this systematic review addressed the importance of modifiable factors, namely parenting styles and feeding practices, on children's body weight. Although theoretically feeding styles may be more difficult to modify than individual feeding practices, there is no evidence thus far to prove that feeding styles are stable and set. In contrast, the findings that mothers with higher education levels have different feeding styles compared to less educated mothers (e.g., Saxton et al., 2009) suggests that environmental factors, potentially including educational interventions, could influence parents' feeding styles. Future research could explore if and how feeding styles can be modified through intervention.

### Future work

One of the key messages from this review is that the ways in which parents feed their children may be influenced by child characteristics such as food approach or avoidance tendencies. However, most studies to date have been conducted cross-sectionally, making it difficult to draw conclusions about the directionality of the associations between feeding practices and child characteristics. Longitudinal studies are required to ascertain the long term, reciprocal nature of parenting, feeding styles, practices, and child characteristics on child BMI. Studies which include measures of child appetitive traits, temperament as well as parental eating behaviors are important so that parenting and feeding styles are understood within the broader context of the parent-child interaction. Therefore, developing measures to assess both child and parent eating behaviors and mealtime interaction is crucial for future research. By better understanding the roles of feeding styles and feeding practices and their interactions with other family factors, clinicians will be better able to counsel families in adopting appropriate feeding practices.

Most of the studies have relied on parent report of their feeding practices and direct observation has been mostly restricted to small scale laboratory-based studies. The development of new technologies for collecting video data through the internet may present opportunities for researchers to film meals in more natural settings. User-friendly webcams or smartphone cameras used during family meal occasions provide a novel platform to complement questionnaire studies to assess parental styles, feeding styles, and feeding practices in the home or other more natural environments.

Culture, ethnicity and SES have been associated with parenting and feeding behaviors (Tovar et al., 2013), however, only a limited number of studies have included participants from diverse backgrounds. As levels of obesity are inversely associated with SES, it is crucial to include more families from less affluent populations. At the same time, research conducted in diverse cultures and varying locations will enhance our understanding of the risk of obesity globally.

Finally, as much of the research focuses on mothers' interactions with children during mealtimes, studies of fathers are warranted to identify gender differences in parenting, feeding styles, and practices (Tschann et al., 2013; Khandpur et al., 2014). Similarly the role of other carers in the child's life is also important to consider. With many children raised in dual-career or single-parent households, the mother is not necessarily the primary caregiver who is providing the child's meals and these other caregivers may be important targets for future interventions.

To conclude, parenting styles, feeding styles, and parenting practices have been associated with child BMI. Protective effects of authoritative parenting and feeding styles might occur as a function of the limit setting, structure, and guidance applied in feeding situations; the limits are used to assist children in learning about food and setting the foundation for healthy eating. Grolnick and Pomerantz (2009) propose that parents who set limits rather than providing free access to foods at home are offering children guidance and structure. Children may need this level of support and guidance to facilitate the formation of healthy eating habits. Specific feeding practices may be used in response to child appetite and temperament; and so child eating traits should be measured in future research. Finally, interventions which are built around authoritative parenting, setting limits, and guiding intake toward healthier foods may help to prevent overweight and obesity.

## Funding

Award number RG.PSYC.103988 to University of Leeds from Nestec SA, Nestlé, Vevey, Switzerland. Support for postdoctoral training in systematic research reviews for Dr. Netalie Shloim.

### Conflict of interest statement

The authors declare that the research was conducted in the absence of any commercial or financial relationships that could be construed as a potential conflict of interest.
